# Comprehensive Assessment of Multiple Sclerosis: From Immunotherapy and Immunopathogenesis to Predictive Biomarkers

**DOI:** 10.30699/IJP.2022.541483.2755

**Published:** 2022-08-11

**Authors:** Soheil Vazifedoust, Hadi Esmaeili Gouvarchin Ghaleh, Mostafa Khafaei, Fateme Azemati, Bahman Jalali Kondori

**Affiliations:** 1 *Baqiyatallah Research Center for gastroenterology and Liver Diseases (BRCGL), Baqiyatallah University of Medical Sciences, Tehran, Iran *; 2 *Applied Virology Research Center, Baqiyatallah University of Medical Sciences, Tehran, Iran*; 3 *Human Genetics Research Center, Baqiyatallah University of Medical Sciences, Tehran, Iran.*; 4 *Department of Biology, School of Basic Sciences, Science and Research Branch, Islamic Azad University, Tehran, Iran*; 5 *Department of Anatomical Sciences, Faculty of Medicine, Baqiyatallah University of Medical Sciences, Tehran, Iran *

**Keywords:** Autoimmune disease, Biomarkers, Immunotherapy, Immunopathogenesis, inflammation, Multiple sclerosis, New treatment

## Abstract

**Background & Objective:**

Multiple sclerosis (MS) is an inflammatory neurological disorder that affects the central nervous system (CNS) and causes individuals to experience a variety of cognitive and physical problems. As proven by two decades of clinical experience with immunomodulatory therapies for MS, the disease progresses and relapses through several immunological pathways. New medicines aimed at remyelination and neurodegeneration are being developed; however, they need stronger evidence before being introduced into routine clinical care. The purpose of this study was a thorough assessment of MS immunopathology and predictive biomarkers.

**Methods:**

Immunotherapy, immunopathogenesis, and prognostic biomarkers were all parts of the search method. Only publications in English were considered for inclusion in the study. For that purpose, we went through the current state of knowledge around MS immunopathology and related biomarkers. Immunology, as well as the identification of increased inflammation as an important component of neurodegeneration, shaped our understanding of this disease aetiology. The relevant sources examined covered the years 2015-2021.

**Conclusion:**

We found biomarkers in the cerebrospinal fluid and blood that might be used for the prediction and diagnosis of MS, as well as for measuring treatment response and adverse effects. Many variables, including the role of some infectious organisms and the impact of environmental and social factors, might contribute to the immunological dysfunctions seen in MS. Patients with MS may benefit from better therapy options if a better understanding of MS biomarkers and immune response mechanisms would be obtained.

## Introduction

**Autoimmune Disease and Multiple Sclerosis**

**Fig. 1:**
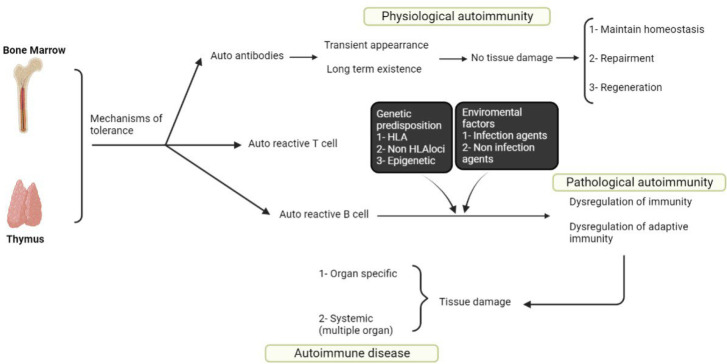
Summary of the autoimmune disease development. In normal individuals, even if there are the strictest controls by peripheral and central tolerance, a few numbers of autoreactive B and T and cells' leak out' into the periphery. They however will not be harmful unless the individual is genetically predisposed to break tolerance and an environmental trigger(s)

In order to defend the host against infectious pathogens, the immune system has evolved to be as diversified as possible. Immune deficiency syndromes, in which one or more parts of the immune system are unable to respond protectively to a pathogen, and autoimmune diseases are the two primary areas of pathology in this pleiotropic immune system (1). The fundamental cause of autoimmune diseases is the failure to distinguish between self and non-self. For example, a person's immune system may be in an "immune tolerance" condition if a certain substance or tissue might provoke an immunological response in that person. Pathological or classical autoimmunity develops when immunological tolerance is broken, and self-reactive cells and autoantibodies are involved in inflammation (Figure 1) (2). 

MS is primarily an inflammatory demyelinating disease of the CNS (central nervous system) with varied histological markers and various clinical symptoms, based on serologic, imaging, genetics, and pathologic findings as well as the patients’ responses to anti-inflammatory therapeutic agents (3). It is most commonly diagnosed between the ages of 20 and 40. However, children might also be affected. MS has also been documented in people above the age of 60. Females are about twice as likely to be affected by this disease as men (4). The common symptoms of MS include tiredness and difficulty walking; visual issues such as impaired vision; bladder control issues; tingling or numbness in various regions of the body; muscular rigidity and spasm; and difficulties with memory, concentration, learning, or planning. In addition to the environmental and genetic variables, the disease pathogenesis includes the development of a pathologic immune-mediated response that causes axonal loss, inflammatory infiltration, and myelin degradation (5). In addition to blood tests, spinal tap (lumbar puncture), MRI, and evoked potential test are other primary methods of MS diagnosis. When it comes to showing the link between relapses and the blood-brain barrier disruption, MRI studies provide the greatest evidence for inflammation-induced relapses. The demyelinated plaque is a key pathological feature of MS, which varies in immunocytological and histological qualities depending on the disease activity (6). Immune-related research must be revised in order to better understand autoimmune diseases, including type 1 diabetes mellitus (T1DM) and MS, according to several academics. In an autoimmune disease, a medicine may be prescribed that is useful for one patient and may be swiftly repurposed to have a significant impact on other patients. Identifying the similarities and differences across numerous autoimmune diseases has the potential to revolutionize the treatment and cure of many diseases in the near future. As a result, research into autoimmune diseases cannot be ignored.

**Search Strategy**

The Medline electronic database, ISI Web of Science, EMBASE, CINAHL, and Scopus were comprehensively searched by two separate researchers through the end of 2021. Immunotherapy, immunopathogenesis, and prognostic biomarkers were all parts of the search method. Only publications in English were considered for inclusion in the study. The project was approved by the Ethics Committee of Baqiyatallah University of Medical Sciences and, accordingly, the ethical code IR.BMSU.REC.1399.509 was obtained.

**New Treatments of MS**

Currently, there are nine kinds of disease-modifying therapies (DMTs). The mechanisms of action, effectiveness, mode of administration, and adverse effects of these drugs differ. There are now several therapy options, and further treatments have been licensed by the FDA (Food and Drug Administration) (16). Although treatment selections should be adjusted to the specific needs and preferences of each patient, there are two main treatments: de-escalation strategy and early, very effective therapy. After starting with lower-to-moderate-efficacy DMTs (e.g., injectable or oral) and experiencing a relapse, the patient's medication may be increased to more effective options (e.g., monoclonal antibody) in a de-escalation strategy (17).

**Table 1:** Summary of the present information about MS

Feature	Mechanism of Disease	Ref
Autoimmunity	MS is a cell-mediated autoimmune disease directed against CNS myelin antigens involving CD8+ and CD4+ cells. There is an enhancing or secondary role for Autoantibodies. The body of normal individuals possesses Autoreactive T cells against myelin elements, and it does not develop the disease in these individuals. It even could provide protective properties for the brain. With the induction of pathogenic Th17- and Th1-type and CD8 myelin autoreactive T cells, MS is developed.	
Infection	Myelin-reactive pathogenic T cells could be induced by Infectious agents. The related possible mechanisms are cross-reaction with the central nervous system's myelin antigens, activating an extended autoreactive immune repertoire, or a self-limited infection of the brain releasing myelin antigens. A lasting brain viral infection or transmissible agent is not the cause of MS.	
Genetics	The risk factors of MS development include MHC and non-MHC genes. Immune repertoire is determined by MHC genes, while tolerance and regulatory mechanisms in MS are determined by non-MHC genes that both of them are defective.	
Environment	The risk of MS development and its course would be increased by Environmental factors, including low UV radiation exposure, low vitamin D, cigarette smoking, EBV exposure, and obesity.	
B cells	B cells centrally play a role in MS. Similar to T cells, anti- and pro-inflammatory B cell subgroups are available. These cells work as the main antigen-presenting cells in relapsing MS, driving pathogenic T cells. B cells in progressive MS are an enhancer of the compartmentalized central nervous system responses achieved via secreted factors and lymphoid follicles.	
Microbiome	The function of T cell is regulated by the microbiome in the body, which is composed of pathogenic microbial and protective components that have a crucial role in MS since they establish immune set points and secrete metabolites.	
Relapsing MS	Immune cells migrating into the CNS drive Relapsing MS. It has been shown that there are some effective therapies for treating relapsing MS (reducing relapses and new MRI lesions). These treatments work on these pathways: reducing function and/or the number of effector cells, increasing function and/or the number of regulatory cells, and preventing cell traffic to the CNS.	
Progressive MS	Mechanisms of Progressive MS are of immune-dependent and immune-independent types. The brain establishes an innate immune response in mechanisms dependent on the immune system. Such response includes macrophages, lymphoid follicles, microglia, and B cells. Also, chronic activation of innate cells and peripheral T cells could be involved. In immune-independent mechanisms, we observe oxidative stress, ion imbalance, and mitochondrial injury. The available treatment does not effectively target these processes.	
Autoantigen	The provoking autoantigen in MS is not known. Nevertheless, with the diagnosis of MS, there is not any singly single autoantigen for targeting since reactivity is spread to other organ-specific antigens, similar to what happens in type 1 diabetes. Therefore, in antigen-specific treatment, bystander suppression should be employed or presented as a preventative strategy for vulnerable individuals.	
Therapy	**MS has a heterogeneous nature. Each effective treatment has responders and non-responders. If the treatment is initiated sooner in the course of the disease, the probability of the therapy's efficacy increases. Effective therapy requires pulse or constant treatment and, eventually, combined treatment. The keystone of MS immunotherapy is identifying MRI and immune biomarkers; thus, no evidence of disease activity (NEDA) would be achieved.**	

Since older drugs have a well-documented safety record, this strategy is often used. Some individuals may be well on their first DMT; however, as the condition progresses, they will need a change in treatment. Clinical relapses and/or new lesions on an MRI (magnetic resonance imaging) are the most prevalent indicators of the disease activity in patients with breast cancer. An even tougher aim of no evidence of disease activity (NEDA) has been proposed by some. Clinical relapses, disability increase, and MRI activity are all included in NEDA-3, whereas brain volume loss is included in NEDA-4 to account for the degenerative process (18). Not yet utilized in clinical practice, NEDA has been considered the desired method. The threshold for de-escalation has been decreased due to the introduction of newer DMTs. Yet, it is reliant on the practitioners' and patients' comfort in using the drugs and the availability of support services for the treatments (e.g., infusion facilities), and the costs. The advantage of therapeutic de-escalation is that it minimizes the risk. Still, the issue is that undertreatment of disease activity might lead to the buildup of impairment and disease progression (19). A viable alternative is to begin treatment with very successful therapy. Subgroup analysis and observational studies have shown that beginning with an early DMT, ideally, after the first clinical episode, leads to improved long-term clinical results. Preventing unnecessary delays in diagnosis and treatment was a primary purpose of the 2017 revision of McDonald criteria. Natalizumab, rituximab, ocrelizumab, and alemtuzumab are among the most effective DMTs (20). Higher effectiveness comes at the cost of greater risk of infection, autoimmune disease, and cancer, as well as a lack of long-term safety evidence for many of these drugs. A range of prognostic markers and shared decision-making between the patient and provider are being used in clinical practice to help patients choose between treatment options. Male gender, older age at presentation, increased severity and frequency of relapses, a higher burden of the spinal cord and infratentorial lesions, increased T2 lesion burden, increased contrast-enhancing lesion burden, and increased brain atrophy are some of the demographic and disease characteristics that may indicate a more severe course (21).

**Immunology of MS**

**1. Effector T Cells in MS**

**Fig. 2:**
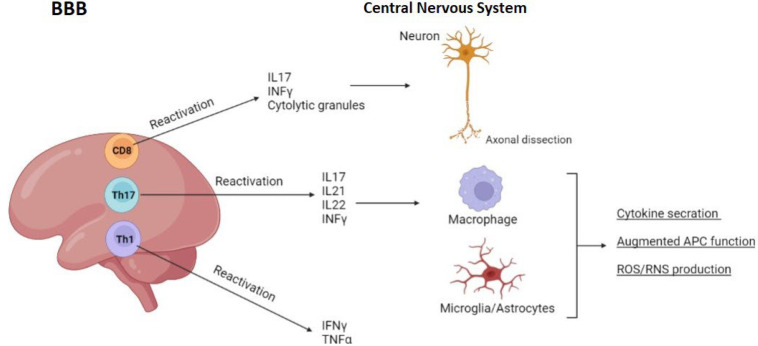
Functions of central and peripheral nervous system activated T cells in MS pathogenesis

After crossing the blood-brain barrier (BBB), peripherally activated T lymphocytes enter the CNS, where they undergo further activation and secrete specific cytokines to carry out their effector functions. T helper 1 (Th1) cells and Th17 lineages release cytokines that define their respective lineages: TNF (tumor necrosis factor) and IFN (interferon)-, and IL (interleukin)-21, IL-22, and IL-17 (22). They can also produce IFN-, which contributes to the pathogenicity of the bacteria in question. IFN and IL-17 can also be produced by CD8+ effector T cells. Antigen-presenting cells (APC) operate better when activated by this cytokine, and the formation of reactive oxygen and nitrogen species (ROS and RNS), as well as the generation of cytokines, is also increased (23). Axonal dissection is caused by CD8 T lymphocytes, which are able to produce distinct cytolytic granules. These FoxP3+ regulatory T cells (Tregs) can be found in the CNS or the peripheral nervous system, along with regulatory B (Breg) cells, natural killer cells (NK), and regulatory T cells (Tregs) (Figure 2) (24).

**Fig. 3:**
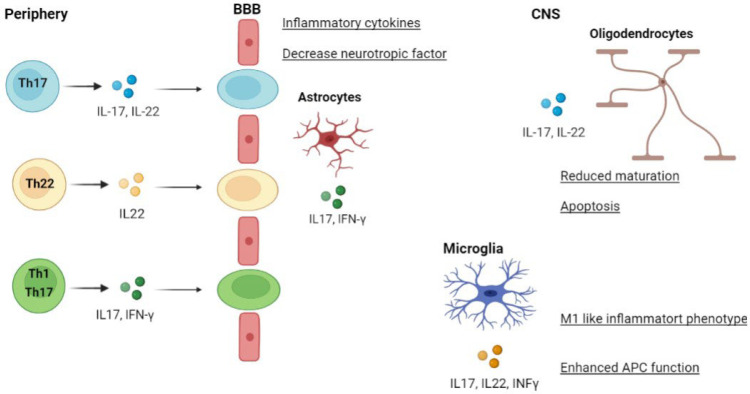
Pathogenic T helper (Th) cell subsets in MS

The self-reactive T cells from the peripheral lymph nodes cross the BBB to reach the CNS (25). T cells regulate the function of CNS-resident cells (oligodendrocytes, astrocytes, microglia) by enhancing the production of inflammatory cytokines, APC activities, and apoptosis (Figure 3) (26).

**2. Pathogenic Function of B Cells in MS**

**Fig. 4:**
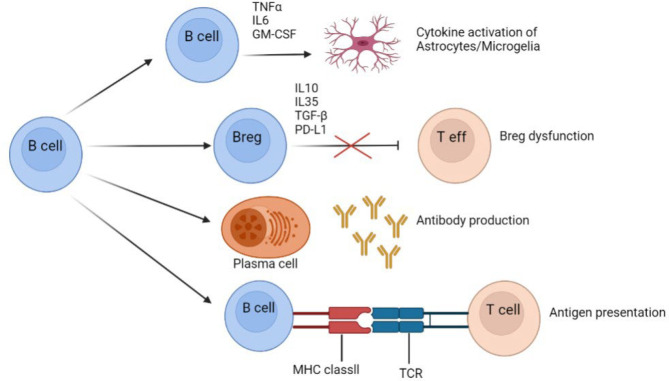
The Pathogenic Roles of B Cells in MS

In terms of major histocompatibility complex (MHC) class II molecules, B cells supply antigens for CD4 T cells activation, which contributes to an inflammatory environment. Breg cells have the ability to inhibit effector T cells via IL-35, IL-10, PD-L1, or TGF-b, or they may be dysfunctional in MS. B cells in the CNS and the peripheral nervous system can create autoantibodies (27). They can cross the BBB and form ectopic germinal centers similar to follicles in the CNS that are categorized and function independently of the peripheral nervous system. B cells can develop and grow into antibody-secreting plasma cells within these follicles in the CNS. Additionally, these cells produce the pro-inflammatory cytokines of TNF-, GM-CSF, and IL-6, which cause CNS inflammation (Figure 4) (28).

**3. Progressive MS Mechanisms**

**Fig. 5:**
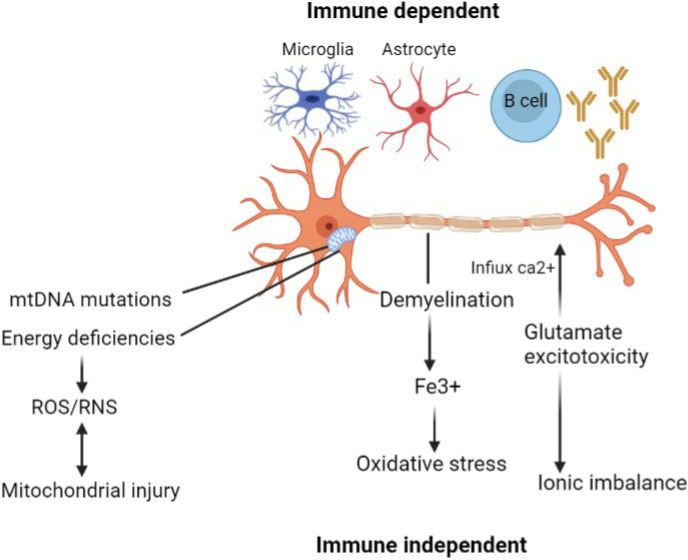
Mechanisms of progression of MS

Progressive MS is associated with the immune cells in the CNS, such as astrocytes and microglia, as well as B cells from ectopic germinal centers. Multiple diseases can be triggered by immune-dependent disease components, which can later transform into immune-independent and self-maintaining damaging mechanisms in the cells (29). An increase in ROS/RNS generation is hampered by an energy deficit resulting from mitochondrial damage caused by a reduced respiratory chain activity and mutations in mitochondrial DNA (mtDNA). Active demyelination areas contribute to oxidative stress, while glutamate-stimulating toxicity occurs in the calcium influx into neurons, which causes ionic imbalances in neurons. Neurodegeneration and axonal atrophy (30) can be caused by both immune-independent and immune-dependent pathways (Figure 5).

**4. Immunotherapy of MS**

Several clinical studies targeting the immune system have been conducted in MS. The clinical aims of immunotherapy vary per disease type (31). Among the therapeutic aims are increasing acute recovery, reducing relapses, and slowing disease progression. The ultimate objective of MS therapy is to treat the patients early enough to arrest the progression. Examples of immunotherapy are modifying lymphocyte traffic, extracorporeal evacuation of serum factors or cells, and modification of antigen-specific cells (32). Nonspecific immunosuppression is becoming more effective through its toxicity limits its use. Developing nontoxic therapies, extending clinical trials, and developing a laboratory monitor for the disease will be required as immunotherapy for MS progresses in future. Given the current good immunotherapy benefits, it is feasible that future immunotherapies may be effective (33).

**Biomarker of MS**

- Individualized treatment decisions may be made utilizing molecular biomarkers, which are critical components of personalized medicine. The ideal biomarker possesses high specificity and sensitivity and a cost-effective, straightforward, non-invasive, and reproducible detection method (34). Currently, by utilizing many well-known biomarkers, it is possible to facilitate MS prediction and diagnosis, as well as risk assessment for adverse effects and treatment response tracking. The IgG index and oligoclonal bands, neutralizing antibodies against IFN- and natalizumab, anti-AQP-4 antibodies, anti-VZV, and anti-JCV antibodies are all examples of these biomarkers. Additionally, there are other interesting biomarker possibilities, such as CHI3L NFL, whose validity should be confirmed in future studies (35). Nonetheless, long-term research through large cohorts is important to further investigate the therapeutic use of biomarker candidates. While some progress has been made in this area, there is still a need for biomarkers that enable reliable prediction of therapeutic response before treatment begins and a tailored therapy (36). As a result, novel biomarkers in the field of MS should continue to be discovered and validated. The biomarkers with (+++) characterization were selected from each group and summarized for the benefit of the readers: *Diagnosis of MS*: The potential biomarkers panel should involve HLA-DRB1* characterization, KFLC and/or CSF OCB IgG, CSF MRZ reaction, MRI with contrast-enhancement, Eps, and serum vitamin D levels (37).
- *Phenotypical expression of MS*: The potential biomarkers panel should chiefly contain antibodies against confrontational epitopes of MOG and MBP, rMOG index, HLA-DRB1* characterization, and IL-6 serum levels. Moreover, measurement of UCCA atrophy and DTI abnormalities could be useful (38).
- *Demyelination-neuroinflammation-relapse*: The potential biomarkers panel should certainly contain CSF MBP-Glutamate-NF-L and CCR2(+) CCR5(+) T-cells, contrast-enhanced T1 MRI lesions, serum levels of IFN-*γ*-TNF-*α*-IL-6-vitamin D, and TOB-1-Fetuin-A-*α*B-crystalline expression. CSF sICAM-1, AR, and CSF sVCAM-1 are acceptable indicators for BBB disruption. Reliable information can be obtained from MRS in studying demyelinating lesions (39).
- *Axonal loss-neurodegeneration*: Nogo-A and NAA are among the major potential biomarkers. Besides, valuable information can be obtained by RNFL and T1 black holes. DTS can be even more helpful in future (40).
- *Prognosis-disability progression*: Among valid prognostic biomarkers of CIS conversion to CDMS are KFLC, OCB IgG, MRZ reaction, NF-H-tau combination, TOB-1 expression, and higher deteriorating rates of gray-matter atrophy. NF-H, NAA, and combined EP' sufficiently are reflective of progression of disability in MS, and GFAP has the same function in NMO. Prognostic information can be obtained from diffusion tensor imaging (DTI) abnormalities for disability progression and relapse (41).
- *Therapeutic response*: Levels of vitamin D and HLA-DRB1* polymorphisms should be regarded as therapeutic outcome biomarkers for IFN-B. RNFL can be considerably presented in the future (42).
- *Differentiation from NMO*: When a differential diagnosis is needed, MRS findings should be beneficial. CSF GFAP, AR, and CSF NAA can also be helpful when NMO is possible (of course, apart from antiAQP4) (43).

**5. Progressive MS Treatment**

Numerous studies around DMT have failed to demonstrate efficacy in progressive MS, and readers are referred to recent reviews for further information on these trials. By describing the pathophysiologic pathways of disease progression versus its relapse, it becomes clear why these studies failed (44). Generally, the immunomodulatory therapy for progressive MS has been somewhat successful since it was applied to cohorts that were in the early stages of progressive MS and still had an inflammatory component (45). It should be noted that anti-CD20 therapy with ocrelizumab was successful in phase III clinical trial of patients with primary progressive MS who had it for less than 15 years and had an Expanded Disability Status Scale (EDSS) score of greater than or equal to 5.0, while those with relapsing-remitting, progressive relapsing, or secondary progressive disease were excluded. As a result, the FDA authorized ocrelizumab for the treatment of primary progressive MS (46).

## Conclusion

Since our understanding of MS has grown tremendously, we have seen a rise in MS medicines, particularly those that target inflammation and relapse. Significant advances have been made in MS identification, measurement, and modification of aberrant immune responses. MS is now acknowledged as primarily an autoimmune disease and not a long-term infection, despite the possibility that an infectious agent triggers the disease. Anti-TNF- therapy was shown to have no beneficial effect; however, anti-CD20 treatment had a significant impact. It needs the development of innovative methods for targeting CNS compartmentalization. Ultimately, the disease may be averted if the environment causes MS in susceptible individuals. In certain circles, immunization against the Epstein-Barr virus or the administration of vitamin D to children at high risk of developing MS is considered an effective first line of defense. Anti-inflammatory therapies that target the immune system in adolescents or children can also prevent the development of MS. Although MS is a disease that has been studied for decades, a substantial portion of it remains unexplained. Biomarker discovery for surrogacy is, thus, an ongoing challenge. It is necessary to perform further research on MS biomarkers to make a more accurate and earlier diagnosis and to develop a quicker, more personalized, and more focused treatment strategy at the lowest possible cost.

## Conflict of Interest

The authors report no conflicts of interest.

## Funding Statement

This study was fully sponsored by Baqiyatallah Research Center for gastroenterology and Liver Diseases (BRCGL), Baqiyatallah University of Medical Sciences, Tehran, Iran.

## Ethical Statement

The study protocol was reviewed and approved by the ethics committee of the Baqiyatallah University of Medical Sciences (IR.BMSU.REC.1399.509).
